# Merits of Level III Axillary Dissection in Node-Positive Breast Cancer: A Prospective, Single-Institution Study From India

**DOI:** 10.1200/JGO.18.00165

**Published:** 2019-02-27

**Authors:** Shalaka Joshi, Jarin Noronha, Rohini Hawaldar, Girish Kundgulwar, Vaibhav Vanmali, Vani Parmar, Nita Nair, Tanuja Shet, Rajendra Badwe

**Affiliations:** ^1^ Tata Memorial Hospital Parel, Mumbai, Maharastra, India

## Abstract

**PURPOSE:**

A complete axillary lymph node (ALN) dissection is therapeutic in node-positive breast cancer. Presently, there is no international consensus regarding anatomic levels to be addressed in complete axillary dissection. We assessed the burden of disease in level III axilla.

**MATERIALS AND METHODS:**

A prospectively maintained database was assessed for 1,591 consecutive patients with nonmetastatic breast cancer registered at Tata Memorial Center, Mumbai, between January 2009 and December 2014.

**RESULTS:**

A median of four (zero to 20) level III ALNs were dissected and a median of two (one to 17) nodes were positive. A total of 27.3% (434 of 1,591) patients had level III ALN metastasis, and 4.7% of patients had positive interpectoral nodes. Some 53.2% of patients had level III metastases in the presence of four or more positive level I and II ALNs. A total of 9.4% of patients had level III involvement when one to three ALNs were positive in level I and II (*P* < .001). Some 53.2% of patients had level III metastases in the presence of four or more positive level I and II ALNs. On logistic regression analysis, four or more positive ALNs in level I or II (*P* < .001), inner/central quadrant tumor location (*P* = .013), and perinodal extension (*P* < .001) were associated with level III ALN involvement. At a median follow-up of 36 months, the disease-free survival was significantly worse for level III ALN metastases on univariate analysis (*P* < .001). On multivariate Cox regression analysis, histologic grade (*P* = .006), four or more positive ALNs (*P* < .001), hormone receptor status (*P* < .001), and tumor size (*P* = .037) were independent prognostic factors for disease-free survival.

**CONCLUSION:**

The axillary nodal burden is high in patients with breast cancer in developing countries like India. One of two women with four or more positive level I and II ALNs may have residual disease in level III if it is not cleared during surgery. Intraoperative interpectoral space clearance should be considered in the presence of either palpable interpectoral lymph nodes or multiple positive ALNs.

## INTRODUCTION

Surgery is the mainstay of treatment of nonmetastatic breast cancer. Mastectomy and breast conservation surgery are proven to have equivalent outcomes in carefully selected patients. It is necessary to stage the axilla for planning appropriate adjuvant treatment. A complete axillary dissection is usually carried out in most node-positive cases. A clinicoradiologically node-negative axilla still has a 30% to 40% possibility of harboring occult metastatic disease.^1^ A sentinel node biopsy or low axillary sampling is necessary to stage the axilla in clinically node-negative cases.^2,3^ In the event of negative axillary lymph nodes (ALNs) on SNB or axillary sampling, one can forgo a complete axillary dissection, with an acceptable false-negative rate of 10%.^4^ Limiting the extent of axillary dissection significantly reduces the morbidity in node-negative patients.^5^ The current practice is to complete axillary dissection in the presence of clinically positive ALNs or positive sentinel nodes.^6^ Recently, the ACOSOG Z11 study demonstrated that observing the axilla is noninferior to a standard axillary dissection, even in the presence of one to three positive ALNs.^7^ In addition, the After Mapping of the Axilla Radiotherapy or Surgery (AMAROS) study has also shown axillary irradiation to be noninferior to axillary dissection. Both studies predominantly included a group of low-risk, postmenopausal hormone-sensitive patients with inherent good prognosis.^8^ Therefore, the applicability of these studies in the Indian scenario is questionable, because only a small proportion of our patients fit the criteria where a complete axillary dissection can be avoided.

Anatomically, the axillary space is divided into three levels by the pectoralis minor muscle. The dissection of level III ALNs, located between the costoclavicular ligament of Halsted and the medial border of pectoralis minor, is associated with a slightly longer surgical time and associated morbidity.^9^ There is no international consensus on the anatomic levels of axilla to be addressed as a part of routine axillary dissection. National Comprehensive Cancer Network guidelines recommend to clear level I and II of the axilla only.^6^ Our institutional policy is to complete the axillary dissection up to level III in all patients with proven positive axillary nodes. The objective of this study was to determine the extent of level III ALN involvement and to ascertain the merits of doing a level III ALN dissection.

## MATERIALS AND METHODS

We reviewed our prospectively maintained database of 1,591 consecutive patients with nonmetastatic breast cancer who underwent up-front surgery at a single high-volume, tertiary care oncology institution, Tata Memorial Center, between January 2009 and December 2014. Patients were evaluated by a multidisciplinary team and underwent mastectomy or breast conservation surgery on the basis of patient choice and disease characteristics. Axillary levels I, II, and III were dissected and sent for pathologic evaluation separately. Details of their histopathological records were retrieved from the electronic medical records.

### Axillary Dissection

Patients with clinicoradiologic positive ALNs or positive nodes on axillary sampling (as determined on frozen section or final histopathology reporting) underwent removal of level I to III ALNs up to the costoclavicular ligament of Halsted. Interpectoral nodes were removed separately. The apical or level III ALNs were addressed via an interpectoral approach by identifying the space between pectoralis major and minor muscles and retracting the pectoralis minor muscle laterally or by a subpectoral approach.

### Statistical Analysis

Baseline clinicopathologic factors of the cohort were reported as numbers and percentage. Univariate analysis was performed using Pearson chi-square or Fisher’s exact test to look for association between level III ALN metastases and other categorical variables. Multivariate analysis was done by logistic regression to identify independent predictors of level III ALN involvement. A receiver operating characteristic curve was plotted to identify the number of ALNs in level I and II to predict level III involvement. The disease-free survival (DFS) was estimated using a Kaplan-Meier curve. The log-rank (Mantel-Cox) test was used to study the impact of prognostic factors on DFS. Multivariate Cox regression analysis was carried out to study the independent prognostic factors affecting DFS. A test was statistically significant if the two-sided *P* value was ≤ .05. Data were analyzed using SPSS version 21.0 (IBM, Armonk, NY) for Windows.

## RESULTS

In our series of 1,591 patients, 889 patients (55.9%) underwent modified radical mastectomy and 702 (44.1%) underwent breast conservation surgery. A total of 98% of patients had infiltrating ductal carcinoma, and 80.4% had grade III tumors. The mean tumor size was 3.2 cm. The tumors were located in the outer quadrant (780 [49%]), inner quadrant (314 [19.7%]), and central/multicentric (433 [27.2%]). A total of 67.3% were hormone-sensitive tumors (estrogen receptor and/or progesterone receptor positive), and 21.6% were human epidermal growth factor receptor 2 positive.

All patients had positive ALNs, with 897 patients (56.4%) having one to three node–positive and 694 (43.6%) having four or more node–positive ALNs. The median total ALNs harvested was 19 (three to 74), of which a median of three (one to 43) ALNs were positive. The details of clinicopathologic characteristics and other demographic features are listed in Table 1. In level I and II axillary nodes, 933 patients (58.6%) had one to three positive nodes, and 648 (40.7%) had four or more positive nodes. Ten patients (0.7%) had skip involvement of level III, with negative level I and II ALNs. Level III ALNs were positive in 27.3% (434 of 1,591) of the patients. A median of four (zero to 20) ALNs were harvested in level III, and a median of two (one to 17) were positive with disease involvement.

**TABLE 1:**
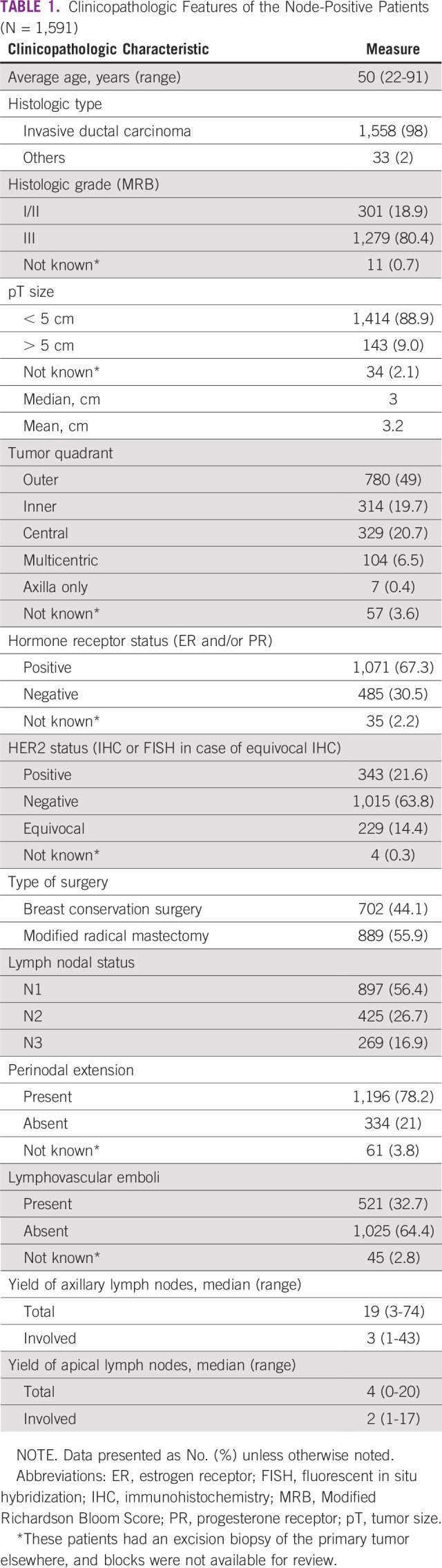
Clinicopathologic Features of the Node-Positive Patients (N = 1,591)

### Interpectoral Lymph Node Status

In 52.3% (835 of 1,591) of patients, an interpectoral lymph node dissection was carried out separately. In 104 (12.4%) of these 835 patients, a lymph node was identified on histopathology, while the others had only fibrofatty tissue. Forty (38.4%) of the 104 interpectoral lymph nodes were metastatic. Thus, the overall rate of interpectoral lymph node involvement was 4.7% (40 of 835).

### Predicting Level III Positivity

**FIG 1:**
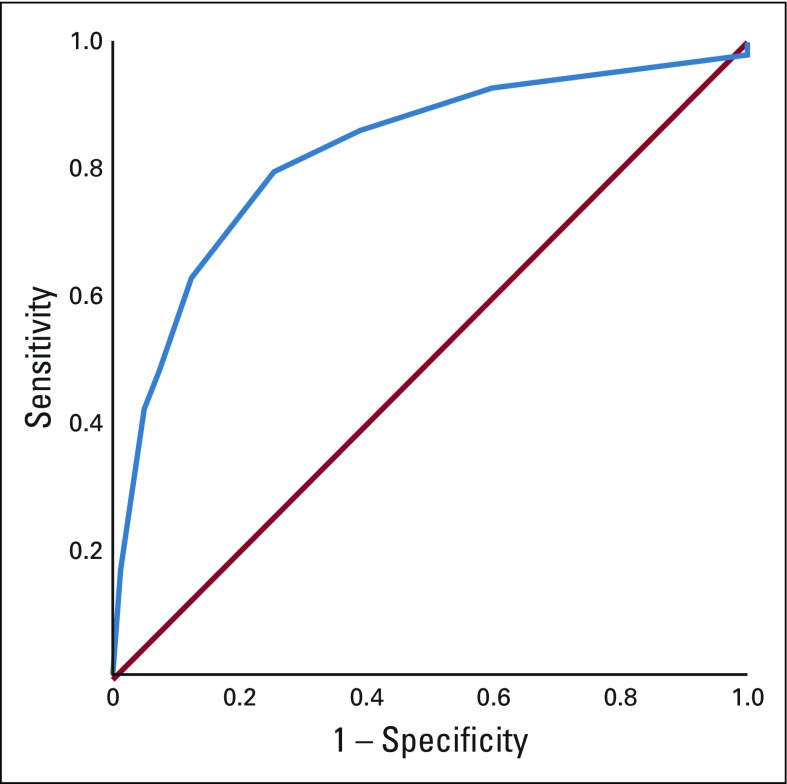
Receiver operating characteristics curve to obtain the cutoff number of axillary lymph nodes involved in level I and II for best predicting level III involvement.

We plotted a receiver operating characteristic curve to identify the number of positive level I and II ALNs that best predicted level III ALN metastases (Fig 1). At a cutoff of 3.5 LNs, the sensitivity, specificity, and positive and negative predictive values for apical ALN positivity were 79.5%, 73.8%, 53%, and 91% (Table 2). For practical purpose, we took the lymph node cutoff as four (instead of 3.5) for all further analyses. When four or more ALNs were positive in level I and II, apical ALN metastasis was seen in 53.2% (345 of 648) of patients, whereas only 9.4% (89 of 943) of patients had positive level III ALNs when one to three ALNs were involved in level I and II.

**TABLE 2:**
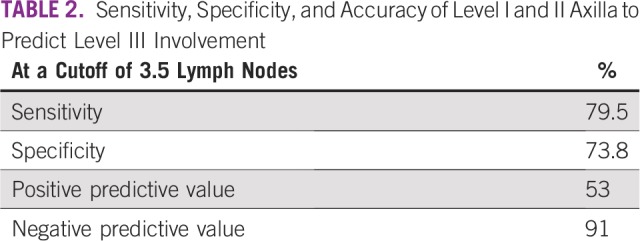
Sensitivity, Specificity, and Accuracy of Level I and II Axilla to Predict Level III Involvement

On univariate analysis (χ^2^/Fisher’s exact test), tumor size greater than 5 cm (*P* =.024), histologic grade III (*P* = .034), human epidermal growth factor receptor 2–positive status (*P* = 0.007), inner/central tumor quadrant location (*P* = 0.002), four or more positive ALNs in level I/II (*P* < .001), presence of perinodal extension (PNE; *P* < .001), and presence of lymphovascular space invasion (LVI; *P* < .001) were significantly associated with level III ALN metastases. Age, histologic grade, and hormone receptor status did not correlate significantly with level III involvement (Table 3).

**TABLE 3:**
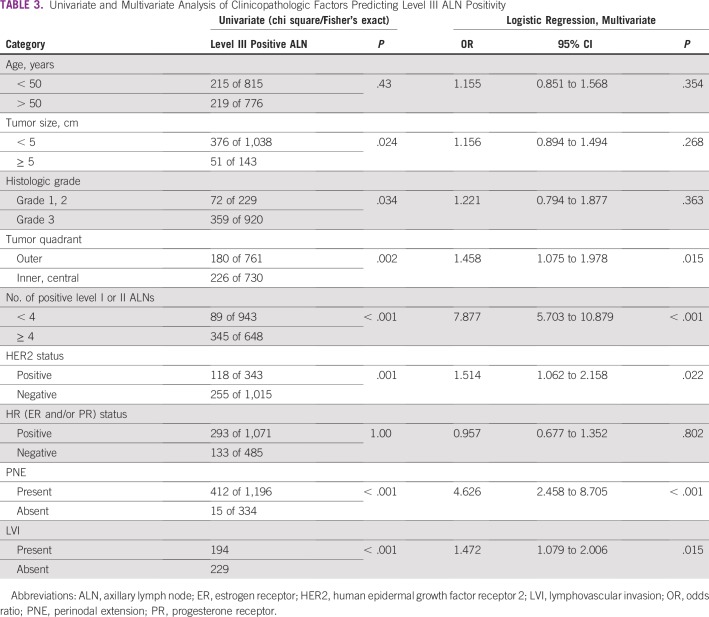
Univariate and Multivariate Analysis of Clinicopathologic Factors Predicting Level III ALN Positivity

On logistic regression analysis, four or more positive ALNs in level I and II (odds ratio [OR], 7.88; 95% CI, 5.70 to 10.88; *P* < .001), inner/central quadrant tumor location (OR, 1.46; 95% CI, 1.07 to 1.98; *P* = .013), and PNE (OR, 4.63; 95% CI, 2.46 to 8.70; *P* < .001) along with LVI (OR, 1.47; 95% CI, 1.08 to 2.00; *P* = .015) were other clinicopathologic features significantly associated with level III ALN positivity (Table 3).

### Survival Analysis

**FIG 2:**
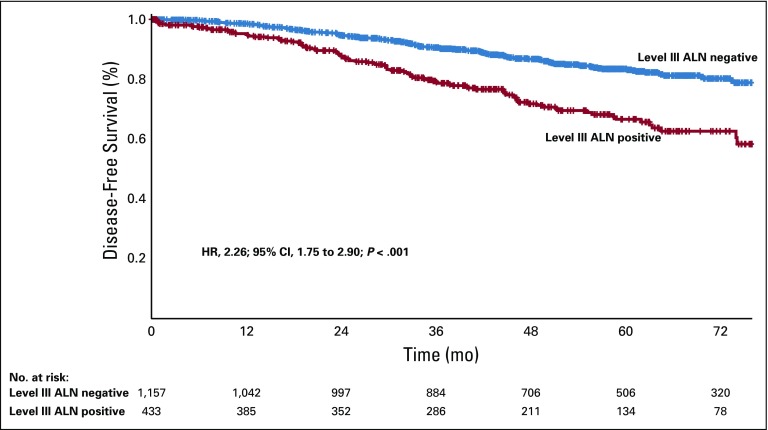
Level III axillary lymph node (ALN) positivity as a prognostic marker for disease-free survival in patients with node-positive breast cancer undergoing up-front surgery. HR, hazard ratio.

At a median follow-up of 36 months, DFS was significantly worse for patients with level III ALN involvement (76% *v* 87.1% in patients with no level III ALN metastases; *P* < .001; Fig 2). On univariate analysis, all factors except age and tumor quadrant had a significant impact on DFS (Table 4). On a multivariate Cox regression analysis, histologic grade (hazard ratio [HR], 2.12; 95% CI, 1.24 to 3.64; *P* = .006), four or more positive ALNs (HR, 2.02; 95% CI, 1.41 to 2.90; *P* < .001), hormone receptor status (HR, 1.82; 95% CI, 1.32 to 2.49; *P* < .001), and tumor size (HR, 0.62; 95% CI, 0.40 to 0.97; *P* = .037) were independent prognostic factors for DFS. Level III involvement was not an independent poor prognostic factor (HR, 1.30; 95% CI, 0.92 to 1.85; *P* = .138) in the multivariate analysis.

**TABLE 4:**
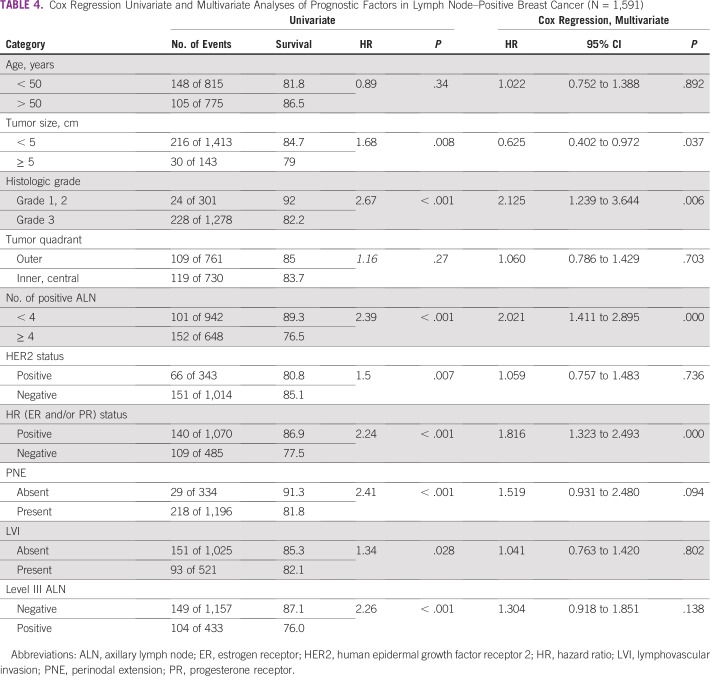
Cox Regression Univariate and Multivariate Analyses of Prognostic Factors in Lymph Node–Positive Breast Cancer (N = 1,591)

## DISCUSSION

Breast cancer surgery has seen a gradual transition from radical mastectomy to breast conservation.^10,11^ Over time, surgical management of the axilla has also seen a conservative shift from complete axillary dissection to sentinel node biopsy.^3,12^ In Milan, a mathematical model constructed using 1,446 patients’ data predicted that to not leave behind residual disease in 90% of patients, a minimum of 10 ALNs had to be dissected.^13^ Hence, in the TNM staging, a minimum of 10 ALNs were believed to be essential for accurate staging of axilla. Clinical examination of the axilla is notoriously inaccurate in staging, with a 30% false-positive rate and a 45% false-negative rate.^14^ In the absence of metastases in a sentinel lymph node biopsy or low axillary sampling, avoiding compete axillary dissection is now accepted as the standard of care.^3^ There are fewer adverse effects with sentinel node biopsy as compared with complete axillary dissection in a node-negative axilla.^3,5,15,16^ The widely acceptable false-negative rate of 10% has not led to a detriment in overall survival in randomized controlled trials.^3,4^ It should be noted that the absolute number of axillary recurrences, albeit small, doubled in the no axillary dissection arm in the NSABP (National Surgical Adjuvant Breast and Bowel Project ) B-32 study.^3^ We routinely carry out low axillary sampling in node-negative axilla, a procedure similar in principle to sentinel node biopsy, with a proven comparable false-negative rate.^2^

ALN metastasis is considered the most important prognostic factor for early breast cancer, and the prognosis worsens as the number of positive lymph nodes increases.^17^ The therapeutic role of axillary dissection has been questioned by many, and some authors have labeled it as only a staging procedure for prognostication and planning appropriate adjuvant therapy.^13^ NSABP B-04 was the first randomized study that reported no survival advantage with axillary dissection.^18^ However, the axillary recurrence rate in the no axillary treatment arm was 19%. A number of other studies have suggested better local control with complete axillary dissection, which amounts to an improvement in survival.^19,20^ The Early Breast Cancer Trialists’ Collaborative Group has reported one life saved for every four local recurrences avoided.^21^ The therapeutic advantage of complete axillary dissection in breast cancer has been proven for node-positive patients.^22^ In a Bayesian meta-analysis of six randomized controlled trials, prophylactic axillary lymph node dissection had an average overall survival benefit of 5.4% (95% CI, 2.7% to 8.0%; probability of survival benefit > 99.5%).^20^ In patients with a positive sentinel node, the American Society of Clinical Oncology does recommend a complete axillary lymph node dissection.^23^ The National Comprehensive Cancer Network recommends routine level I clearance and palpation of level II and III for presence of nodes.^6^ In a recent Cochrane review, treatment with less axillary surgery (sentinel node biopsy or axillary sampling) was associated with a reduced overall survival as compared with a complete axillary lymph node dissection (HR, 1.09; 95% CI, 1.01 to 1.17; 18 studies; 6,478 patients).^24^ They concluded that limited axillary surgery is acceptable only in pathologically proven node-negative axilla.

In our analysis, we found a high incidence of level III ALN involvement (ie, 27.3% of all node-positive patients). The probability of positive level III ALNs was as high as 53.2% when four or more LNs were positive in level I and II (*P* < .001). Almost 50% of patients with high level I and II nodal burden run the risk of having residual disease in the axilla after surgery if level III is not addressed. In a similar study from Turkey, 31% of the 86 patients undergoing a mastectomy had positive level III lymph nodes.^14^ Other studies have found level III LN positivity ranging from 15% to 59%.^25,26^ Skip metastases to level III ALNs are documented to occur in 0% to 15% of patients; we found the rate of skip metastases to be 0.7%.^25,27^ In our study, four or more ALNs in level I and II, inner/central quadrant tumor location, poor histologic grade, and presence of PNE and LVI were associated with higher level III ALN positivity. Few studies have tried to demonstrate the correlation between level III positivity and other clinicopathologic factors. In a study by Chua et al,^28^ 320 patients were evaluated. Involvement of lymph nodes in level III was observed in 22 patients (7%), and 51 patients (16%) had four or more positive nodes. Palpability of ALNs, pathologic tumor size, and LVI were significantly associated with level III involvement and four or more positive nodes by univariate and multivariate analyses. Up to 42% of patients had involved level III ALNs when four or more ALNs were positive, similar to the 53.2% seen in our study. Khafagy et al^25^ reported that 53.5% of patients had level III ALN involvement when a lower level had nodal metastases. Veronesi et al^26^ also reported an incremental risk of level III involvement with increasing number of positive lower-level nodes. The level III involvement was 8%, 25.3%, and 65.8%, respectively, when 1, 2, and 4 or more ALNs were positive in level I. In another study from China by Fan et al,^29^ the incidence of residual positive nodal disease in level III was 9% (47 of 521), even after preoperative neoadjuvant chemotherapy in stage I and II breast cancer. They showed a significantly worse distant DFS when level III ALNs were involved (84.9% and 91.6% in level III positive and negative groups, respectively; *P* = .011).

We found overall interpectoral node positivity to be 4.6%. If a node was found on histopathology, it was positive 38.4% of the time. Other studies have shown interpectoral node positivity ranging from 0.1% to 14%.^25,30,31^

Involvement of level III ALNs is a poor prognostic factor in our study. Patients with level III ALN involvement had a worse DFS (87.1% *v* 76%; *P* < .001). Previous studies have shown a similar worse survival outcome with involvement of level III nodes.^17,32^ Our study is the largest prospective series addressing separate level III ALN dissection in a node-positive axilla. However, one of the main shortcomings of our study is the impact of level III dissection on survival; we cannot comment on this, because we do not have an arm for comparison where axillary level III dissection was not carried out. This was studied in a randomized manner in two studies from Japan. Tominaga et al^9^ divided 1,209 patients undergoing mastectomy for early breast cancer to axillary dissection up to level II or resection of pectoralis minor muscle and axillary dissection up to level III. The 10-year DFS rates were 73.3% and 77.8%, respectively (HR, 0.94; *P* = .666). In another trial by Kodama et al,^33^ 514 patients were divided to undergo breast conservation surgery and mastectomy and further stratified to undergo either level I or level I to III axillary dissection. There were no significant differences in the 10-year overall and DFS rates in the two groups. The authors concluded that compared with exclusive level I clearance, level III dissection resulted in a longer operation time and greater blood loss but did not improve the survival rate. The disadvantage of these two studies is that they included both node-negative and node-positive patients together, potentially diluting the benefit of a complete axillary clearance in node-positive patients. Hence, the impact of level III clearance in node-positive axilla has never been studied in a systematic manner, and it is unlikely that there will now be a randomized study in this setting. We have previously described an interpectoral approach to perform a level III lymph node dissection by retracting the pectoralis minor muscle laterally.^34^ The surgical time as well as blood loss, nerve injury, and vascular injury have been minimal with our technique. We had previously published an analysis of 148 patients who underwent revision surgery for incompletely performed initial breast resections elsewhere. One hundred twenty-three out of 148 patients (83.1%) had residual disease in the axilla, and 64 (52.03%) had positive ALNs.^35^ This could potentially have an impact on survival as well. An R0 surgical resection is currently the most standard method of achieving optimal local control of disease in breast cancer. Adjuvant radiation is an adjunct to complete surgical clearance for better locoregional outcomes and for overall survival.^21^ We emphasize the role of complete surgical resection in breast cancer, especially in a lower middle-income country like ours, where the stage at presentation and the incidence of axillary nodal metastases is high

Surgical resection is the mainstay of treatment of nonmetastatic breast cancer, and a complete surgical resection is necessary for optimal local control. In a lower middle-income country like India, there is a higher stage at presentation and a higher incidence of axillary nodal involvement. We recommend a routine axillary clearance up to level III in node-positive axilla, especially when multiple lower-level axillary nodes are involved. Intraoperative interpectoral space palpation with clearance should be considered in the presence of either palpable interpectoral lymph nodes or multiple positive ALNs.

## Data Availability

The following represents disclosure information provided by authors of this manuscript. All relationships are considered compensated. Relationships are self-held unless noted. I = Immediate Family Member, Inst = My Institution. Relationships may not relate to the subject matter of this manuscript. For more information about ASCO's conflict of interest policy, please refer to www.asco.org/rwc or ascopubs.org/jco/site/ifc. No potential conflicts of interest were reported.
